# A review of the Holarctic genus *Tmeticus* Menge, 1868 (Araneae, Linyphiidae), with a description of a new genus

**DOI:** 10.3897/zookeys.59.508

**Published:** 2010-10-01

**Authors:** Yuri M. Marusik, Seppo Koponen

**Affiliations:** 1 Institute of Biological Problems of the North, RAS, Portovaya Str. 18, Magadan 685000, Russia; 2 Zoological Museum, University of Turku, FI-20014 Turku, Finland

**Keywords:** spiders, Erigoninae, Palaearctic, Nearctic, new synonym, new combination

## Abstract

Eight species attributed to Tmeticus are reviewed; five are redescribed and illustrated: Tmeticus affinis (Blackwall, 1885), Tmeticus bipunctis (Bösenberg & Strand, 1906), Tmeticus nigriceps Kulczyński, 1916, Tmeticus ornatus (Emerton, 1914) and Tmeticus tolli Kulczyński, 1908. The new genus, Paratmeticus gen. n. is erected for Tmeticus bipunctis, and a new combination is established: Paratmeticus bipunctis (Bösenberg & Strand, 1906), **comb. n.** Three species names: Gongylidium vile Kulczyński, 1885, **syn. n.**, Tmeticus difficilis Kulczyński, 1926, **syn. n.** and Tmeticus dubius Kulczyński, 1926, **syn. n.**, are synonymized with Tmeticus tolli Kulczyński, 1908. Although Gongylidium vile has date priority over Tmeticus tolli it is synonymized because of the lack of usage. Three species from Japan attributed to Tmeticus: Tmeticus neserigonoides Saito & Ono, 2001, Tmeticus nigerrimus Saito & Ono, 2001 and Tmeticus vulcanicus Saito & Ono, 2001 are not related to Tmeticus affinis, the type species of the genus, and their affinities remain unclear. The male of Tmeticus nigriceps is described for the first time.

## Introduction

Tmeticus is a small Erigoninae genus with nine species restricted to the Holarctic Region (Platnick 2010). Members of this genus can be easily recognized by their elongate male palp with a small bulbus and a ventral tooth on the patella. Only the type species of the genus, Tmeticus affinis (Blackwall, 1855), occurs both in the Palaearctic and Nearctic Regions. One species, Tmeticus ornatus (Emerton, 1914), is restricted to the Nearctic Region. All other species have been recorded from Asia. The highest species diversity of the genus is in Japan, as a result of three recently described species.

While studying the Siberian and Far Eastern Linyphiidae, we encountered certain difficulties in identifying Tmeticus species. Only two of the four species occurring in northern Asia, Tmeticus affinis (Blackwall, 1885) and Tmeticus tolli Kulczyński, 1908, were properly illustrated. Thus, the main purposes of this study are to provide diagnostic illustrations for each Asian species and to describe a new genus.

## Material and methods

Pictures of the general appearance and copulatory organs were made using an Olympus SZX16 stereomicroscope, with an Olympus E-520 camera, and prepared using CombineZM software. Photographs were taken in dishes of different sizes with paraffin in the bottom. Different sized holes were made in the bottom to retain the specimens in the desired position. Scanning electron micrographs were made using a SEM JEOL JSM-5200 scanning microscope. SEM and digital photographs were made in the Zoological Museum, University of Turku. The terminology of the copulatory organs follows Hormiga (2000). The smallest and biggest specimens are reported, all measurements are in millimetres.

### Abbreviations

IBPNInstitute for Biological Problems of the North, Russian Academy of Sciences, Magadan (curator Yu.M. Marusik).

PSUDepartment of Zoology, Perm State University (curator S.L. Esyunin).

ZMMUZoological Museum of the Moscow State University (curator K.G. Mikhailov).

ZMUTZoological Museum, University of Turku (curator S. Koponen).

### Tmeticus

Menge, 1868

#### Type species.

Tmeticus leptocaulis Menge, 1868 (= Tmeticus affinis (Blackwall, 1855)).

#### Diagnosis.

Males of this genus are easily recognized by possessing a mastidion (large tooth on frontal part of chelicera) and by their elongate palp with patella longer than cymbium, ventral terminal tooth on patella, and thin bulbus (as wide as terminal part of tibia). Females are recognized by their flat epigyne without a cavity. Males may be confused only with the trans-Palaearctic Hylyphantes graminicola (Sundevall, 1830) because it also has a mastidion and a patellar tooth. However, the males of Hylyphantes have shorter palp, undivided embolic division and screw-like embolus.

The females of Tmeticus may be confused with those of several genera, such as Oedothorax Bertkau, 1883 or with Donacochara speciosa (Thorell, 1875). However, Oedothorax females have a different colour pattern, and Donacochara speciosa is notably larger.

#### Description.

Small to medium-sized (2.5–4.1), light to dark-coloured erigonines. Male carapace unmodified and without sulci, it may be uniformly coloured or with a darker cephalic region. Abdomen unmodified, dark, of uniform colour. Male chelicera modified by possessing a mastidion (*Ma*, promarginal tooth). Maxilla with apical-retrolateral spine. Tibial spines 2-2-1-1. TmIV present. TmI 0.65–0.8. Male palp elongate. Femur, patella and tibia longer than wide. Patella with conical, ventral terminal tooth (*Tt*). Tibia with two apophyses (*Ta*). Paracymbium large, with or without (Tmeticus affinis) distinct apical pocket. Tegulum with distinct sac (*Ts*) and large (Tmeticus tolli) or small protegulum (*Pt*). Radix with straight apical process (*Ap*), tailpiece (*Tp*) without extension, embolus (*Em*) short and straight, or long and forming a semicircle; embolic membrane (*Me*) large. Epigyne without distinct fovea or openings. Median (=dorsal, *sensu* Hormiga 2002) plate plain or with ridges.

#### Composition.

According to Platnick’s (2010) catalogue eight species are listed in this genus: Tmeticus affinis (Blackwall, 1855) (Holarctic), Tmeticus bipunctis (Bösenberg & Strand, 1906) (Far East Asia), Tmeticus neserigonoides Saito & Ono, 2001 (Japan), Tmeticus nigerrimus Saito & Ono, 2001 (Japan), Tmeticus nigriceps (Kulczyński, 1916) (Northern Siberia), Tmeticus ornatus (Emerton, 1914) (USA & Canada), Tmeticus tolli Kulczyński, 1908 (Siberia) and Tmeticus vulcanicus Saito & Ono, 2001 (Japan). In fact, there are three more species names within this genus: Tmeticus difficilis Kulczyński, 1926, Tmeticus dubius Kulczyński, 1926 and Gongylidium vile Kulczyński, 1885. Of these, the first two are listed under Centromerus, and the last one under Oedothorax. These three names were included in Tmeticus by Holm (1973) and Eskov (1994) but were considered as synonyms of Tmeticus tolli.

On the basis of the present study, we conclude that Tmeticus encompasses four species: Tmeticus affinis, Tmeticus nigriceps, Tmeticus ornatus and Tmeticus tolli. A new genus has been erected for Tmeticus bipunctis. Tmeticus neserigonoides might be correctly placed in this genus, but as we failed to re-examine its specimens, we treat it as *incertae sedis* (see below). Two other Japanese species belong elsewhere, but their correct assignments require further study.

#### Comments.

Tmeticus is unusual in the Erigoninae because all its species can be recognized by their carapace colour pattern. Three sibling species: Tmeticus nigriceps, Tmeticus ornatus and Tmeticus tolli cannot be recognized by their embolic division, but the females of these species have distinctly different epigynes.

#### Interrelationships.

Tmeticus affinis differs from the three other species by the shape of the paracymbium, the straight embolus and the high protegulum with papillae. It also possesses a different type of the tibial apophysis, not originating at the terminal edge of the tibia as in other Erigoninae and other Tmeticus, but slightly aside of the edge.

#### Relationships.

In general appearance, male palp structure and cheliceral dentition, the members of this genus are similar to Hylyphantes graminicola, but the latter has a different type of embolic division and epigyne. When Wiehle (1956) described Donacochara speciosa (Thorell, 1875) he compared it with Tmeticus affinis. Both species have a long palp, small bulbus, and the chelicera of the male has a mastidion. The embolic division in both species is rather similar, but the radical process and the embolus proper occupy different positions.

The embolic division of Tmeticus is similar to those in Phaulothrix hardyi (Blackwall, 1850) (cf. Millidge 1977: f. 18), Lophomma punctatum (Blackwall, 1841)(Fig. 58) or members of Oreoneta Chyzer & Kulczyński, 1894 (Fig. 57). All these genera have a more or less straight, two-armed embolic division (embolus proper + anterior radical process), with the embolus proper situated more dorsally than the process. All three genera have a wide embolic membrane.

Millidge (1977) placed Tmeticus in a separate nominative group with Ostearius Hull, 1911, Donacochara Simon, 1884, Eboria Falconer, 1910 and Sciastes Bishop & Crosby, 1938. Hormiga (2002) placed Tmeticus close to a very heterogeneous group of the higher Erigoninae that includes such unrelated genera (in terms of the structure of the embolic division) as Walckenaeria Blackwall, 1833 (twisted radix), Oedothorax, Entelecara Simon, 1884, Gonatium Menge, 1968 and others.

Judging from the drawings (Figs 35.110, 35.111 in Draney and Buckle 2005), Tmeticus can be related to the Nearctic Nanavia monticola Chamberlin & Ivie, 1933. The latter species seems to have been mistakenly considered a synonym of Leptorhoptrum robustum (Westring, 1851) (see Platnick 2010). Both genera and species were synonymized by Eskov and Marusik (1994) on the basis of a comparison of Leptorhoptrum robustum and the poor figures of Nanavia monticola. Nanavia monticola has a very long palpal tibia, and the paracymbium and embolic division are very similar to those of Tmeticus affinis. The relationships of the two genera and taxonomic status of Nanavia Chamberlin & Ivie, 1933 are outside the scope of this study and will be considered elsewhere.

#### Key to Tmeticus species

The males of Tmeticus nigriceps and Tmeticus ornatus cannot be distinguished.

**Table:** 

1.	Carapace uniformly coloured (Figs 10–11, 25–26)	2
–	Cephalic part darker than red/orange thoracic part (Figs 21–24)	3
2.	Carapace red, orange/yellow; occurs in Siberia and the Far East	Tmeticus tolli
–	Carapace reddish brown, with slightly lighter posterior part (Figs 10–11), tibia with two small claw-like apophyses (Figs 1–2, 5, 7), median plate of epigyne square-shaped (Figs 4, 8–9, 19); distributed throughout the Holarctic	Tmeticus affinis
3.	Cephalic part dark brown (Figs 23–24), epigyne with extended median plate (Figs 18, 43–44); occurs in the tundra zone of Siberia	Tmeticus nigriceps
–	Cephalic part brown (Figs 21–22), epigyne without extension (Figs 17, 47, 48); occurs in southern Canada and the northern United States	Tmeticus ornatus

## Species survey

### Tmeticus affinis

(Blackwall, 1855)

Figs 1 11 19 49 61

Neriene affinis Blackwall 1855: 121 (D♂).
Tmeticus affinis : Wiehle 1960: 411, f. 751–756 (♂♀).
Tmeticus affinis : Merrett 1963: 410, f. 83A-C (♂).
Tmeticus affinis : Millidge 1977: 37, f. 150 (♂).
Tmeticus affinis : Roberts 1987: 44, f. 12e (♂♀).
Tmeticus affinis : Millidge 1993: 147, f. 32 (♀). For a complete set of references see Platnick (2010).

#### Material examined.

FINLAND: 2♀ (ZMUT), Turku Ruissalo, 14.11.1966 (M. Saaristo); 2♀ (ZMUT), Turku Ruissalo, sea shore litter, 27.10.1966 (M. Saaristo); 1♂ (ZMUT) Turku Hirvensalo Illoinen, 30.5.1966 (P.T. Lehtinen); 1♀ (ZMUT), Turku Kärsämöki Pomponrahka, 30.05.1967 (M. Saaristo); 1♀ (ZMUT) Pori Yyteri, 16.10.1966 (M. Saaristo); 1♀ (ZMUT), Pudasjärvi Hirvaskoski, 12.08.1959 (P.T. Lehtinen); 1♂ (ZMUT), Kuusamo Torankijärvi, 7.7.1966 (M. Saaristo); 1♀ (ZMUT), Kajaani, Koutaniemi, 16.07.1972 (P.T. Lehtinen); 1♂ (ZMUT) Inari Repojoki, 9.7.1961 (O.V. Lindqvist); 1♀ (ZMUT) Utsjoki Kevo, birch forest on lake shore, 20.06.-20.07.1970 (E.T. Linnaluoto). RUSSIA: ***Krasnoyarsk*** Province, 1♂ 2♀ (ZMMU), Mirnoye, Yenisei River left bank, 23.06.1978 (K.Yu.Eskov); 2♀ (ZMMU), Mirnoye, Yenisei River left bank, 27.07.1979 (K.Yu. Eskov). ***Yakutia***, 2♂ 2♀ (ZMUT), El’gyay, big ”alas” pond, 24.07.1977 (S. Koponen); 1♂ (ZMMU), western Yakutia, Kempendyay River 80 km up stream from the mouth, riverside meadow, 1–15.08.1988 (K.Yu. Eskov). ***Kamchatka*** Peninsula, 1♂ 1♀ (IBPN), Talovskoye Lake, Kuyul River, 16.08.1990 (M.B. Skopets). ***Chukotka***: 1♂ (ZMMU), Markovo, July 1986 (G. Chernova). CANADA, ***Alberta***: 1♂ (only the photo provided by D.J. Buckle has been studied), Caribou Mountain Wildlands, Wentzel Lake, 50°02N; 114°28W, sweeping horsetail meadow, 16.07.2003 (T. Johnson).

**Figures 1–4.**
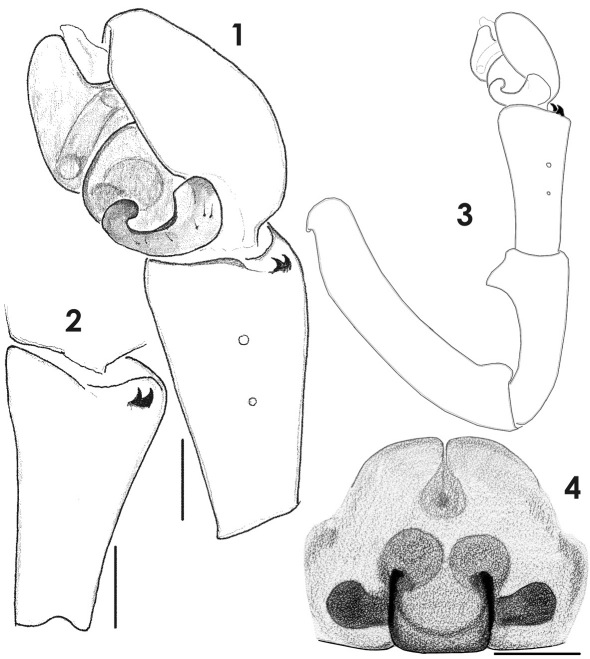
Copulatory organs of Tmeticus affinis. **1** male palp, retrolateral view **2** palpal tibia, dorso-lateral view **3** whole male palp, retrolateral view **4** epigyne, ventral view. (scale bar 0.1 mm).

**Figures 5–9.**
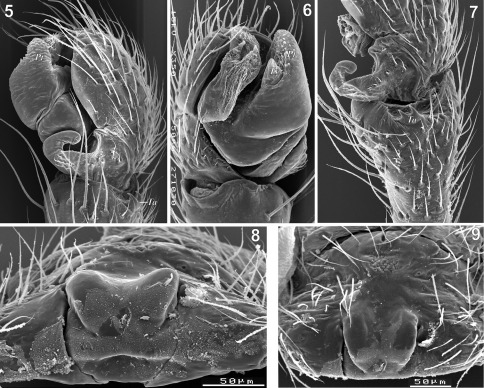
Copulatory organs of Tmeticus affinis. **5** male palp, retrolateral view **6** male palp, prolateral view **7** male palp, dorsal view **8** epigyne, caudal view **9** epigyne, ventral view. Abbreviations: ***Pt*** - protegulum; ***Ta*** - tibial apophysis; ***Ts*** - tegular sac.

**Figures 10–20.**
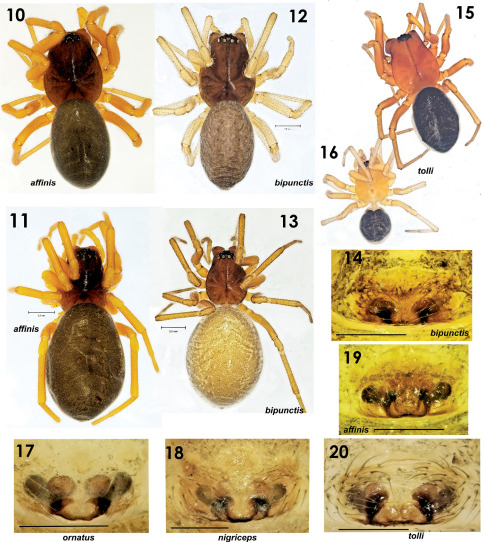
Habitus and epigyne of Tmeticus affinis (**10–11, 19**), Paratmeticus bipunctis (**12–14**), Tmeticus tolli (**15–16, 20**), Tmeticus ornatus (**17**) and Tmeticus nigriceps (**18**). **10, 12, 14–16** male habitus, dorsal view 11, 13 female general appearance, dorsal view **14, 17–20** epigyne, ventral view **15–16** difference in the size between males from the same sample. (scale bar 0. 2 mm, if not otherwise indicated).

#### Diagnosis.

This species is easily recognized by its brownish carapace with a darker cephalic region. Males are easily recognized by their palp, which has a characteristic tibial apophysis and embolic division with the anterior radical process equal in length to the embolus proper (embolus longer than anterior radial process in other species). Females are easily recognized by the shape of the epigyne.

#### Description.

For detailed description see Wiehle (1960). ♀ 2.5–3.0, ♂ 2.5–2.8. TmI 0.65–0.75. Carapace reddish brown, rather dark in males. Cephalic region slightly darker than thoracic, but there is no clear demarcation between the two. Abdomen black. Legs orange-brown. Palp as in Figs 1–3, 5–7, 49, 61; epigyne as in Figs 4, 8–9, 19.

#### Distribution.

This species is known all over Eurasia, from western Europe to Kamchatka. In the Nearctic Region, it has been reported from Alberta (Nordstrom and Buckle 2006).

### Tmeticus tolli

Kulczyński, 1908

Figs 15–16, 20 25 34 50 56

Gongylidium vile Kulczyński 1885: 37, pl. 10, f. 16 (♀), syn. n.
Tmeticus tolli Kulczyński 1908: 15, pl. 1, f. 3, 7–8, 22–23 (♂♀).
Tmeticus difficilis Kulczyński 1926: 50 (♀), syn. n.
Tmeticus dubius Kulczyński 1926: 49 (♀), syn. n.
Centromerus tolli : Sytshevskaja 1935: 90.
Tmeticus tolli : Holm 1973: 89.
Tmeticus tolli : Eskov 1994: 107.
Tmeticus tolli : Hormiga 2000: 56, f. 29A-I, pl. 67A-F, 68A-F (♂♀).
Tmeticus affinis : Marusik and Logunov 1999: 245 (misidentification).

#### Material examined.

RUSSIA: **Krasnoyarsk** Province: 1♂ 2♀ (ZMMU), Mirnoye, Yenisei River left bank, 23.06.1978 (K.Yu. Eskov). **Evenkiya**: 40♂♀ (ZMMU), Taimura River, Neptene River mouth, riparian spruce forest with alder, Summer 1982 (K.Yu. Eskov); 2♀ (ZMMU), Chambe River mouth, meteorological station “Kerbo”, floodplain willow stand, litter, 21.08.1982 (K.Yu. Eskov). ***Khabarovsk*** Province: 2♀ (IBPN), Okhotski Dist., Gyrbykan R. (Ul’ya River basin), 20.08–15.09.1986 (I.D. Sukatcheva); 1♂ 3♀ (IBPN), Khetana River (tributary of Amka River, Ulya River basin), Agust 1985 (V.V. Zherikhin). ***Maritime*** Prov.: 3♂ 2♀ (IBPN), [05], Khanka Lake CW shore, Sosnovy Isl & peninsula nearby, 44°52N; 132°07E, 17.07.1998 (Yu.M. Marusik). 1♀ (IBPN), [03], Khanka Lake, CE shore, 44°39N; 132°34E, 15–16.07.1998 (Yu.M. Marusik). ***Magadan*** Area: 3♂ 2♀ (IBPN), Motykley Bay, 59°30N; 148°50E, Summer 1994 (E. Izergina); 1♂ 1♀ (IBPN), 137th km of Kolyma Hwy, 60°25N; 151°30E, Ola River, valley forest, 28.09.1994 (Yu.M. Marusik); 1♂ 2♀ (IBPN), ca 50 km N of Magadan, Khasyn River, environs of Splavnaya Vil., 28.05.1988 (Yu.M. Marusik); 25♂♀ (IBPN), 30km N of Magadan, Snow Valley Vil., Dukcha River valley, 7.10.1984 (Yu.M. Marusik). ***Sakhalin*** Island: 1♂ 6♀ (IBPN), Okha Dist., Ten’ga River, May 1987 (A.M. Basarukin); 4♀ (IBPN), Tomari Dist., Ainskoye Lake, Ptichya river, 24.05.-10.06.1984 (A.M. Basarukin); 1♀ CE part, Leonidovka River, 8 km SE of Leonidovo Vil., 49°16.506N; 142°58.390E, 9.08.2001 (Yu.M. Marusik). ***Kamchatka*** Peninsula: 2♀ (ZMMU), 40 km from Ust’-Kamchatsk, 09.1973 (A.S. Glikman); 1♂ 4♀ (IBPN), 10–12 km N of Paratunka Vil., Yelizovo Forestry, 53.050°N; 158.225°E, 15–28.07.2004 (A.S. Ryabukhin). MONGOLIA: ***Arkhangai*** Aimak: 2♂ 2♀ (IBPN) [12], Ondrer-Ulaan, Tsakhir, Chulut gorge 48°07N; 100°22E, 2100 m, 10–13.06.1997 (Yu.M. Marusik). ***Central (=Tov)*** Aimak: 1♀ (IBPN), Terelzh Mt., south exposed slope (about 80 km NE of Ulan-Bator, 1988 (S. Heimer).

**Figures 21–26.**
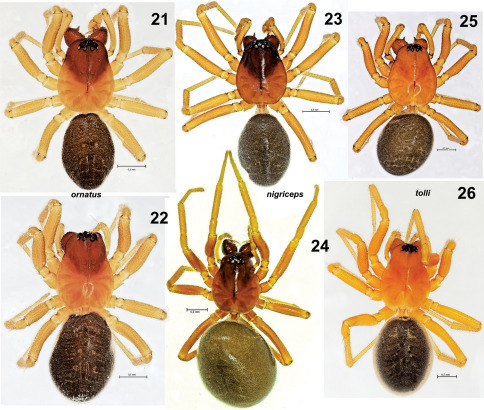
Habitus of Tmeticus ornatus (**21–22**), Tmeticus nigriceps (**23–24**) and Tmeticus tolli (**25–26**). **21, 23, 25** male, dorsal view **22, 24, 26** female, dorsal view.

**Figures 27–31.**
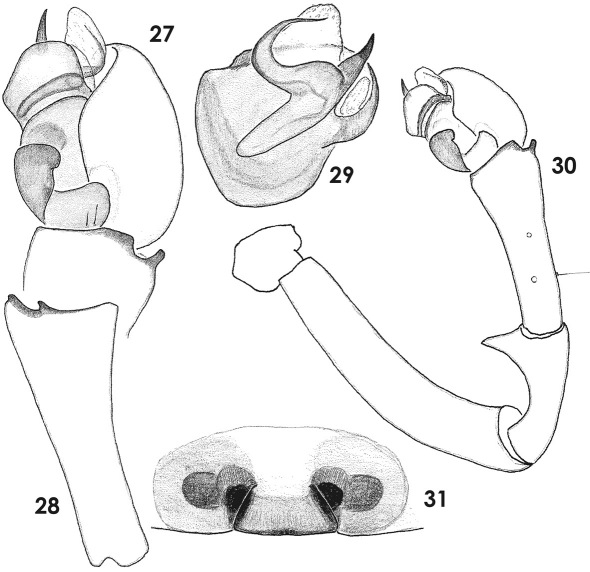
Copulatory organs of Tmeticus tolli. **27** male palp, retrolateral view **28** palpal tibia, dorso-lateral view **29** bulbus, prolateral view **30** – whole male palp, retrolateral view **31** epigyne, ventral view.

**Figures 32–34.**
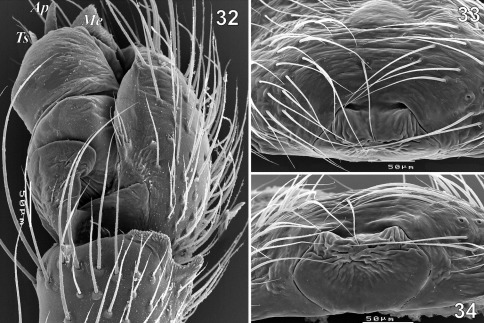
Copulatory organs of Tmeticus tolli. **32** male palp, retrolateral view **33** epigyne, ventral view **34** epigyne, caudal view. Abbreviations: ***Ap*** – anterior radial process; ***Me*** - embolic membrane; ***Ts*** - tegular sac.

#### Diagnosis.

Tmeticus tolliis easily distinguished from the similar Tmeticus ornatus and Tmeticus nigriceps by having a uniformly coloured carapace in both sexes.

#### Description.

Both sexes were described in detail by Hormiga (2000). ♀ 2.8–4.1, ♂ 2.5–3.1. TmI 0.68–0.8. Carapace from orange to pale yellow with black median stripe. Abdomen from light brown to blackish. Legs coloured as carapace. Palp as in Figs 27–30, 32, 50, 56, epigyne as in Figs 20, 31, 33–34.

#### Comments.

Tmeticus difficilis Kulczyński, 1926 was described on the basis of the female holotype from Lake “Kurarotschnoje” (=Kurazhechnoye, ca. 56°10N; 161°45E, collected 9.06.1909) and Tmeticus dubius Kulczyński, 1926 was described on the basis of two females from Lake “Klutschevskoje” (= maybe Klyuchi Vil., c. 60 km from Kurarochnoye Lake). In his descriptions Kulczyński (1926) compared both species with Tmeticus tolli. Both species were transferred to Centromerus (a member of the Micronetinae, a different subfamily) by Reimoser (1919) and this transfer was not contested by Charitonov (1932). Sytschevskaya (1935), who collected in the same places, suggested that both species, in addition to Gongylidium vile Kulczyński, 1885 (from Petropavlovsk-Kamchatski) maybe conspecific with Tmeticus tolli. Holm (1973) agreed with Sytschevskaya (1935). Eskov (1994) and Mikhailov (1997) listed the threespecies as synonyms of Tmeticus tolli, but formal synonymies were not proposed. In addition, Gongylidium vile (listed as Oedothorax vilus in Platnick’s (2010) catalogue has date priority over Tmeticus tolli.

Although Kulczyński (1885) compared Gongylidium vile with European Oedothorax (the epigyne of Tmeticus tolli is very similar to those in Oedothorax), he mentioned the colour of the carapace “*flavido-rufo*” (yellow-red = orange), which is typical for Tmeticus tolli and such coloration is absent from Oedothorax species. Furthermore, no Oedothorax species has been recorded from Kamchatka. In order to retain stability we suggest suppression of the name Gongylidium vile Kulczyński, 1885, because it does not appear in the literature (except for catalogues and nomenclatorial notes), whereas Tmeticus tolli has been used in more than 25 publications by more than 10 different authors during the past 50 years (Holm 1973; Eskov 1988, 1994; Koponen and Marusik 1992; Marusik et al. 1992, 1993, 2002; Marusik 1993, 2005a-b; Rybalov et al. 2002; Hormiga 2000; Tanasevitch 2006; Trilikauskas and Tanasevitch 2006; etc.). We agree with Eskov about the status of these species and here formally propose three new synonymies: Gongylidium vile Kulczyński, 1885, syn. n. = Tmeticus difficilis Kulczyński, 1926, syn. n. and Tmeticus dubius Kulczyński, 1926, syn. n. = Tmeticus tolli Kulczyński, 1908.

#### Distribution.

This species is distributed east of Yenisei (Eskov 1994) to Chukotka and southward to central Mongolia (Marusik and Logunov 1999: sub. Tmeticus affinis), Maritime Province of Russia (present data) and northern Sakhalin (Eskov 1994). Tmeticus tolli also occurs in northeastern China. YM saw one female specimen of this species in the collection of Baoding University (China).

### Tmeticus nigriceps

(Kulczyński, 1916)

Figs 18 23–24 35–44 52 55

Gongylidium nigriceps Kulczyński 1916: 8, pl. 1, f. 10 (♀).
Tmeticus nigriceps : Holm 1973: 89.
Tmeticus nigriceps : Eskov 1994: 107.

#### Misidentifications.

(all refer to Zornella cultrigera (L. Koch, 1879) see Holm (1973):

Gongylidium nigriceps: Tullgren 1955: 355, f. 56 (♀).

Gongylidium nigriceps: Hauge 1969: 6, f. 12 (♀).

#### Material examined.

RUSSIA: ***Arkhangel’sk*** Area: 1♂ (IBPN), Barents Sea, Dolgiy Ilsand, 69°12'N, Summer 2004 (O.L. Makarova). ***Polar Ural***: 1♂ (ZMUT), Oktyabrskij, Ob River shore, Salix viminalis vegetation, 12.-13.7.1994 (S. Koponen); 1♀ (PSU-95), North Ural expedition by Fridolin, sample 36, Sob’ River right bank, 4.07.1925 (V. Fridolin). ***Yamal*** Peninsula: 1♂ 5♀ (ZMMU), Yorkugayakha River, environs of “Canary” trading station, riparian willow stand, 08.07.2002 (D. Osipov); 4♂ 1♀ (ZMMU), south Yamal, Shchuchye Vill, Shchuchya River (A.L. Tikhomirova); 1♂ (PSU-96), South Yamal, Khadyta-Yakha River, meadow valley, pitfall traps, 8.08.1982 (S.L. Esyunin); 1♂ 1♀ (PSU-97), same locality, river bank, drift, 26.07.1981 (S.L. Esyunin). ***Taimyr*** Peninsula: 1♂ 1♀ (ZMMU), Taimyr Reserve, Novaya River, Ary-Mas Site, 25.07.1992 (A.B. Ryvkin); 10♂ 1♀ (ZMMU), SW Taimyr, Nyapan’ Ridge, 70°09N; 87°47E, Carex-moss bog, pitfall trapping, 1–10.08.1999 (D. Osipov); 3♂ (ZMMU), NW shore of Pyasino Lake, 70°04'54N; 87°32'12E, Carex bog with sphagnum tussocks, 10–20.07.1999 (D. Osipov); 3♂ 2♀ NW shore of Pyasino Lake, 70°04'54N; 87°32'17E, Carex bog with sphagnum tussocks, Summer 1999 (D. Osipov); 2♀ (ZMMU), NW shore of Pyasino Lake, Lazannakh Lake, sandy beach, 70°05'47N; 87°26'28E, 1–10.07.1999 (D. Osipov). ***Yakutia***: 2♀ (IBPN), Yana River down flow, environs of Kular Village, 70°35N; 134°34E, grass-herb- Arctagrostis meadow on the former open mine, Summer, 2000 (N.K.Potapova). ***Chukotka***: 2♂ (IBPN), western Chukotka, Chaun River mouth part, 68.810°N; 170.432°E, Summer, 1986 (A.S. Ryabukhin); 1♂ (IBPN), western Chukotka, Chaun Bay, Pucheveyem River mouth, 25.07.1985 (A.S. Ryabukhin); 1♂ (IBPN) Lamutskoye Vil., 65°32'39N; 168°51'08E, along creek, 17.08.1968 (Novikova); 1♂ (IBPN), western Chukotka, Markovo Town, July 1986 (G. Chernova); 1♂ (IBPN), Vulvyveyem River upper flow, Gytlenumkuum Stand, 67°10N; 178°E, 8.06.1988 (Yu.M. Marusik).

**Figures 35–39.**
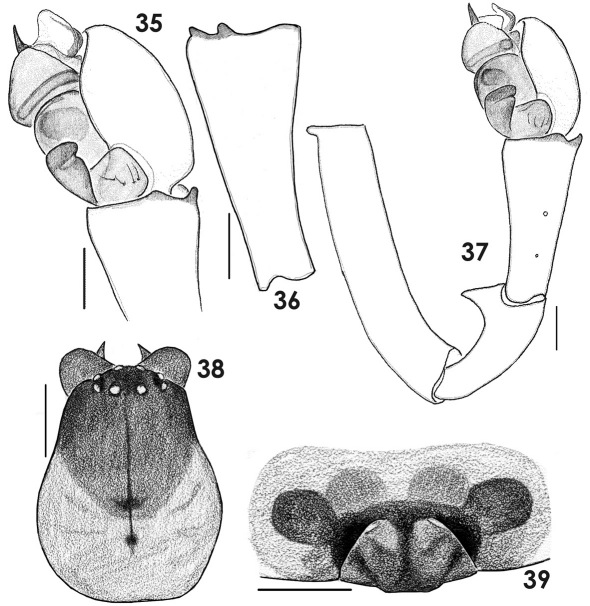
Copulatory organs and male carapace of Tmeticus nigriceps. **35** male palp, retrolateral view **36** palpal tibia, dorso-lateral view **37** whole male palp, retrolateral view **38** male carapace, dorsal view **39** epigyne, ventral view. (scale bar 0.1 mm).

**Figures 40–44.**
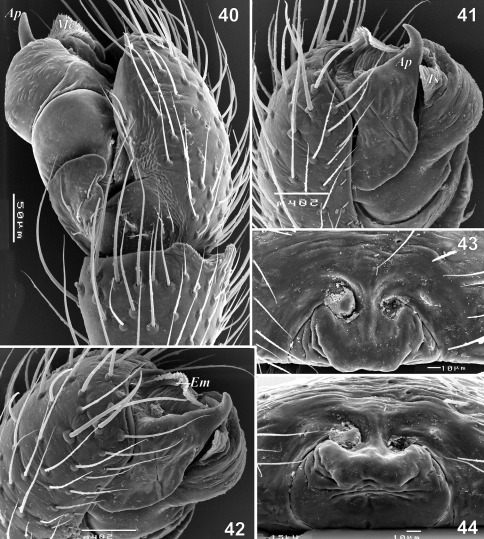
Copulatory organs of Tmeticus nigriceps. **40** male palp, retrolateral view **41** male palp, prolateral view **42** male palp, dorso-prolateral view **43** epigyne, ventral view **44** epigyne, caudal view. Abbreviations: ***Ap*** – anterior radial process; ***Em*** - embolus proper; ***Me*** - embolic membrane; ***Ts*** - tegular sac.

**Figures 45–48.**
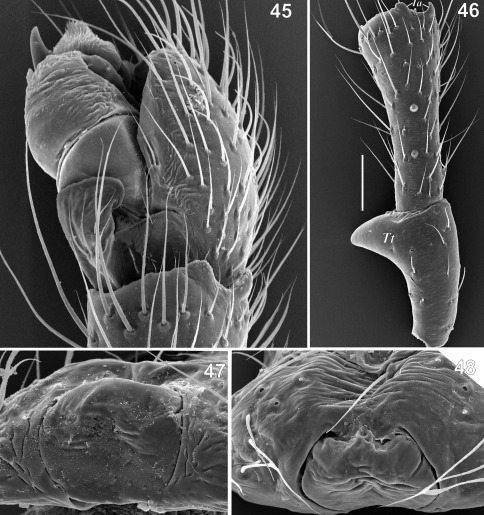
Copulatory organs of Tmeticus ornatus. **45** male palp, retrolateral view **46** male palpal patella and tibia, retrolateral view **47** epigyne, caudal view **48** epigyne, ventral view. Abbreviations: ***Tt*** – patellar tooth.

**Figures 49–53.**
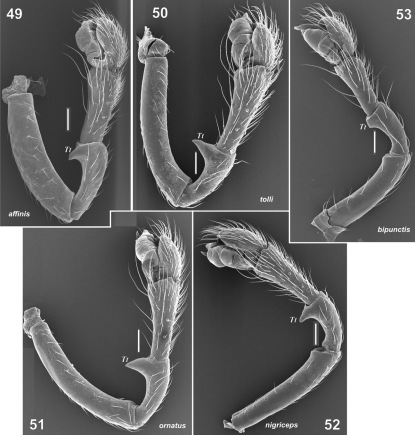
Retrolateral view of the male palp of Tmeticus affinis (**49**), Tmeticus tolli (**50**), Tmeticus ornatus (**51**), Tmeticus nigriceps (**52**) and Paratmeticus bipunctis (**53**). (scale bar 0.1 mm).

#### Diagnosis.

Tmeticus nigriceps is easily distinguished from the other Palaearctic species by the dark cephalic region contrasting with the reddish thoracic area. Only the Nearctic Tmeticus ornatus has a similar colour pattern. The male palp is almost undistinguishable from the Siberian Tmeticus tolli and the Nearctic Tmeticus ornatus. In addition to the carapace pattern, females can be distinguished by the shape of their epigyne.

#### Description.

♀ 2.9–3.3, ♂ 2.3–2.7. TmI 0.69–0.72. Carapace orange with dark, blackish cephalic region (Figs 23–24, 38) and chelicera. Legs orange. Abdomen black. Palp as in Figs 35–37, 40–42, 52, epigyne as in Figs 18, 39, 43–44.

#### Comments.

Holm (1973) re-examined the specimens from Sweden identified and recorded as Gongylidiun nigriceps by Tullgren (1955) and concluded that they were misidentifications of Zornella cultrigera (L. Koch, 1879). The figure of the female epigyne from Norway in Hauge (1969) identified as Gongylidium nigriceps also refers to Zornella cultrigera.

#### Distribution.

This species is known from Dolgiy Island and the Polar Urals to Chukotka (Eskov 1994). It is restricted to the tundra zone.

**Figures 54–61.**
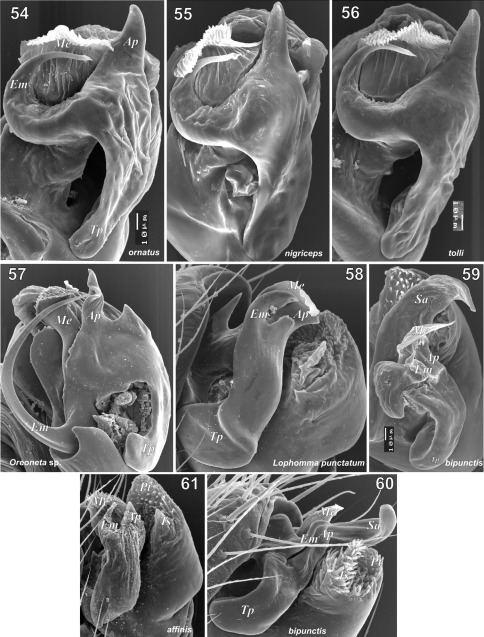
Embolic division of Tmeticus ornatus (**54**), Tmeticus nigriceps (**55**), Tmeticus tolli (**56**), Oreoneta sp. (**57**), Lophomma punctatum (**58**), Paratmeticus bipunctis (**59–60**) and Tmeticus affinis (**61**). Abbreviations: ***Ap*** – anterior radial process; ***Em*** - embolus proper; ***Ma*** – mastidion; ***Me*** - embolic membrane; ***Pt*** -  protegulum; ***Sa*** - distal suprategular apophysis; ***Tp*** - tailpiece; ***Ts*** - tegular sac.

**Figures 62–65.**
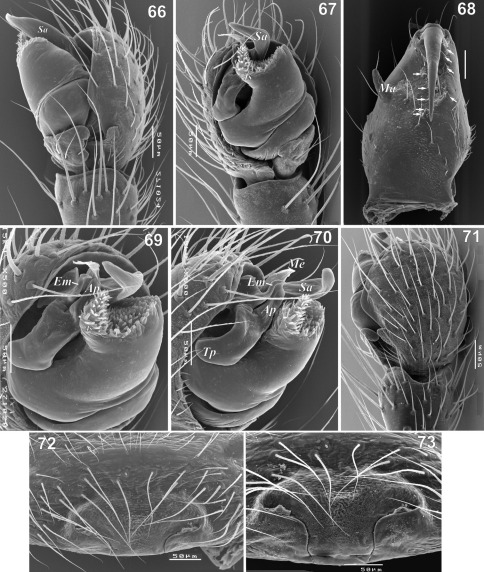
Copulatory organs of Paratmeticus bipunctis. **62** male palp, retrolateral view **63** male palpal tibia, dorso-retrolateral view **64** whole male palp **65** epigyne, ventral view.

**Figures 66–73.**
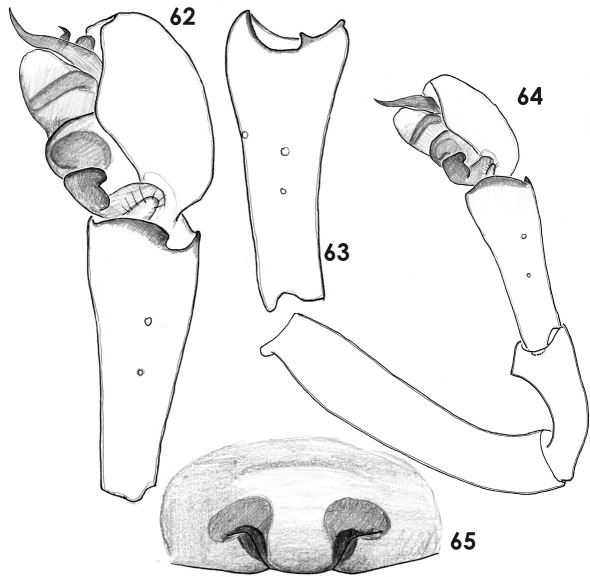
Copulatory organs and male chelicera of Paratmeticus bipunctis. **66** male palp, retrolateral view **67** male palp, ventral view **68** male chelicera inner view **69** male palp, from above **70** male palp, prolateral view **71** male palp, dorsal view **72–73** epigyne, ventral and caudal view. Arrows show the cheliceral teeth. Abbreviations: ***Ap*** – anterior radial process; ***Em*** - embolus proper; ***Ma*** – mastidion; ***Me*** – embolic membrane; ***Sa*** - distal suprategular apophysis; ***Tp*** - tailpiece; ***Ts*** - tegular sac.

### Tmeticus ornatus

(Emerton, 1914)

Figs 17 21–22 45–48 51 54

Gongylidium ornatus Emerton 1914: 263, pl. 8, f. 3 (♂).
Tmeticus ornatus : Bishop and Crosby 1935: 227, pl. 18, f. 22–26 (♂♀).
Tmeticus ornatus : Paquin and Dupérré 2003: 122, f. 1284–1286 (♂♀).

#### Material examined.

CANADA: 4♂ 4♀ (ZMMU), ***Saskatchewan***, Lady Lake, sedge tops – flooded marsh, 13–15.04.1971 (D.J. Buckle); 3♂ 3♀ (ZMUT), same locality, marsh, late April, 1978 (J.V. Buckle).

#### Diagnosis.

Differs from Tmeticus affinis, which also occurs in the Nearctic Region, by the carapace colour (black cephalic region and red-orange thoracic area in Tmeticus ornatus, carapace uniformly brown in Tmeticus affinis). The males are easily separated by their tibial apophyses (one apophysis with a claw-like processes in Tmeticus affinis and two separate apophyses in Tmeticus ornatus); the females have distinctly different epigynes.

#### Description.

♂ 2.5–3.3, ♀ 2.8–35. TmI 0.73–0.78. Carapace orange with darker cephalic region. Abdomen dark. Palp as in Figs 45–46, 51, 54. Epigyne as in Figs 17, 47–48.

#### Distribution.

This species has a trans-Nearctic distribution, recorded from British Columbia to Quebec and south to New York (Buckle et al. 2001). It does not occur north of 55°N and has a more southern distribution in comparison to the Palaearctic Tmeticus affinis, Tmeticus tolli and Tmeticus nigriceps.

## Species Incertae Sedis

The three species from Japan assigned to Tmeticus remain unstudied and belong elsewhere (see ‘Comments’ below).

### Tmeticus neserigonoides

Saito & Ono, 2001

Tmeticus neserigonoides Saito and Ono 2001: 9, f. 15–20 (♂♀).
Tmeticus neserigonoides : Ono et al. 2009: 304, f. 638–642 (♂♀).

#### Comments.

Judging from the available figures, this species might belong in Tmeticus, The male has a long palp with a patellar tooth. However, the chelicera appears to lack a mastidion and the tibial apophyses are absent. Figures of the male palp are unclear, TmI index (0.59) is lower than in Tmeticus species (>0.63).

### Tmeticus nigerrimus

Saito & Ono, 2001

Tmeticus nigerrimus Saito and Ono 2001: 13, f. 26–31 (♂♀).
Tmeticus nigerrimus : Ono et al. 2009: 304, f. 643–647 (♂♀).

#### Comments.

This species is clearly not related to Tmeticus affinis or other members of the genus due to the short palpal patella lacking a tooth in the male, embolic division of a different shape, the relatively long tibial apophysis, lack of a mastidion, epigyne with a septum and some other additional characters. The correct generic placement remains unclear.

### Tmeticus vulcanicus

Saito & Ono, 2001

Tmeticus vulcanicus Saito and Ono 2001: 11, f. 21–25 (D♂♀).
Tmeticus vulcanicus : Ono et al. 2009: 304, f. 648–652 (♂♀).

#### Comments.

This species is clearly not related to Tmeticus affinis or other members of the genus due to the short palpal patella lacking a tooth in the male, embolic division of a different shape (anterior radical process absent), and some other characters. The correct generic placement remains unclear.

### Paratmeticus gen. n.

urn:lsid:zoobank.org:act:3F57381F-374C-4D90-9312-24A8419BF422

#### Type species.

Oedothorax bipunctis Bösenberg and Strand, 1906.

#### Etymology.

Prefix “Para”- indicates the resemblance of this genus to Tmeticus The gender is masculine.

#### Diagnosis.

The new genus is easily distinguished from the similar Tmeticus by lacking distinct tibial apophyses, and in having the papillate tegular sac larger than the protegulum, a slightly twisted embolic division, a sharply pointed embolic membrane and a large distal suprategular apophysis, longer than the embolic division. In contrast to Tmeticus, themedian plate of the epigyne in the new genus is widest in the anterior region, rather than in the posterior region.

#### Description.

Medium-sized erigonine spiders. Uniformly coloured, male carapace without modifications, male chelicera with mastidion, inner row with 4 inner teeth and 5 outer teeth (all smaller than inner teeth). TmI 0.63–0.65. Male palp elongate, with patella as long as tibia, tibia lacks apophyses, distal suprategular apophysis longer than embolic division; embolic division slightly twisted with two arms: anterior radical process and embolus proper; embolus parallel to process with lamellate basal process; epigyne without cavity, median plate widest anteriorly.

#### Composition.

The type species only.

### Paratmeticus bipunctis

(Bösenberg & Strand, 1906) comb. n.

Figs 12–14 53 59–60 62 73

Oedothorax bipunctis Bösenberg and Strand 1906: 162, pl. 12, f. 258 (♀).
Tmeticus japonicus Oi 1960: 152, f. 50–51 (♂).
Tmeticus japonicus : Chikuni 1989: 58, f. 56 (♂♀).
Tmeticus japonicus : Eskov 1994: 107.
Tmeticus bipunctis : Saito and Ono 2001: 7, f. 10–14 (S, ♀).
Tmeticus bipunctis : Ono et al. 2009: 304, f. 634–637 (♂♀).

#### Material examined.

RUSSIA: ***Sakhalin*** Island: 1♂ 4♀ (IBPN), Okha Dist., 5–7 km N of Kolendo Vil., 22–23.08.1991 (A.M. Basarukin); 1♂ 2♀ (IBPN), Pil’tun Bay, 06.-0.7.1991 (A.M.Basarukin); 1♂ 1♀ (IBPN), Okha Dist., Ten’ga River, May 1987 (A.M. Basarukin); 10♂ 26♀ (IBPN), Korsakov Dist., Tunaiga Lake south shore, 26.09.1991 (A.M.Basarukin). ***Kuril*** Isles, 4♂ 3♀ (IBPN), Paramushir Isl. NE shore, environs of Severo-Kuril’sk, 50°40N; 156°06E, 10.08–15.09.1996 (Yu.M. Marusik); 2♂ 1♀ (ZMMU), Iturup Island, Dobroye Nachalo Bay, Lesozavodskoye, mixed forest, 14.08.1994 (K.Yu. Eskov). ***Kamchatka*** Peninsula, 6♂ 2♀ (IBPN), 10–12 km N of Paratunka Vil., Yelizovo Forestry, 53.050°N; 158.225°E, 15–28.07.2004 (A.S. Ryabukhin).

#### Description.

Well described by Saito and Ono (2001) and Ono et al. (2009). Total length: ♂ 2.5–3.2, ♀ 2.8–3.5. Carapace 1.29–1.57 long, 1.0–1.24 wide, slightly larger in males. Chelicera in male with mastidion. TmI 0.63–0.65. Carapace dirty brownish, sternum and chelicerae brown. Abdomen dark grey-blackish. Palp as in Figs 53, 59–60, 62–67, 69–71, epigyne as in Figs 14, 65, 72–73.

#### Distribution.

Kamchatka (south part), 8 islands in Kuril Archipelago (Shikotan, Kunashir, Iturup, Urup, Simushir, Ketoi, Shiashkotan, Paramushir, but seems to occur on all large islands); Sakhalin and Japan (Hokkaido, Honshu and Kyushu).

## Supplementary Material

XML Treatment for Tmeticus

XML Treatment for Tmeticus affinis

XML Treatment for Tmeticus tolli

XML Treatment for Tmeticus nigriceps

XML Treatment for Tmeticus ornatus

XML Treatment for Tmeticus neserigonoides

XML Treatment for Tmeticus nigerrimus

XML Treatment for Tmeticus vulcanicus

XML Treatment for Paratmeticus

XML Treatment for Paratmeticus bipunctis
